# ECDC’s latest publications

**DOI:** 10.2807/1560-7917.ES.2016.21.44.30389

**Published:** 2016-11-03

**Authors:** Eurosurveillance editorial team

**Affiliations:** 1 European Centre for Disease Prevention and Control (ECDC), Stockholm, Sweden

**Rapid risk and outbreak assessments**
Date of publication: 31 October 2016
Zika virus disease epidemic. Ninth update, 25 October 2016 http://ecdc.europa.eu/en/publications/_layouts/forms/Publication_DispForm.aspx?List=4f55ad51-4aed-4d32-b960-af70113dbb90&ID=1595#sthash.FD9TSZIT.dpuf
Date of publication: 27 October 2016
Joint Rapid Outbreak Assessment: Multi-country outbreak of *Salmonella Enteritidis* phage type 8, MLVA type 2-9-7-3-2 and 2-9-6-3-2 infections
http://ecdc.europa.eu/en/publications/_layouts/forms/Publication_DispForm.aspx?List=4f55ad51-4aed-4d32-b960-af70113dbb90&ID=1593
Date of publication: 24 October 2016
Extensively drug-resistant tuberculosis – multi-country cluster, Romania
http://ecdc.europa.eu/en/publications/_layouts/forms/Publication_DispForm.aspx?List=4f55ad51-4aed-4d32-b960-af70113dbb90&ID=1590
Date of publication: 10 October 2016
Outbreak of Rift Valley fever in Niger - Risk for the European Union
http://ecdc.europa.eu/en/publications/_layouts/forms/Publication_DispForm.aspx?List=4f55ad51-4aed-4d32-b960-af70113dbb90&ID=1579

**Scientific advice**
Date of publication: 17 October 2016
Public consultation: Expert opinion on rotavirus vaccination in infancy
http://ecdc.europa.eu/en/publications/_layouts/forms/Publication_DispForm.aspx?List=4f55ad51-4aed-4d32-b960-af70113dbb90&ID=1586

**Evidence brief**
Date of publication: 18 October 2016
Pre-exposure prophylaxis for HIV prevention in Europe
http://ecdc.europa.eu/en/publications/_layouts/forms/Publication_DispForm.aspx?List=4f55ad51-4aed-4d32-b960-af70113dbb90&ID=1587

**Technical documents**
Date of publication: 19 October 2016
Handbook on using the ECDC preparedness checklist tool to strengthen preparedness against communicable disease outbreaks at migrant reception/detention centres
http://ecdc.europa.eu/en/publications/_layouts/forms/Publication_DispForm.aspx?List=4f55ad51-4aed-4d32-b960-af70113dbb90&ID=1588
Date of publication: 14 October 2016
Communication strategies for the prevention of HIV, STI and hepatitis among MSM in Europe
http://ecdc.europa.eu/en/publications/_layouts/forms/Publication_DispForm.aspx?List=4f55ad51-4aed-4d32-b960-af70113dbb90&ID=1584
Date of publication: 12 October 2016
Point prevalence survey of healthcare-associated infections and antimicrobial use in European acute care hospitals – protocol version 5.3
http://ecdc.europa.eu/en/publications/_layouts/forms/Publication_DispForm.aspx?List=4f55ad51-4aed-4d32-b960-af70113dbb90&ID=1581

**Technical reports**
Date of publication: 13 October 2016
External quality assessment scheme for influenza antiviral susceptibility for the European Reference Laboratory Network for Human Influenza – 2015
http://ecdc.europa.eu/en/publications/_layouts/forms/Publication_DispForm.aspx?List=4f55ad51-4aed-4d32-b960-af70113dbb90&ID=1582
Date of publication: 13 October 2016
External quality assessment scheme for detection, isolation and characterisation of influenza viruses for the European Reference Laboratory Network for Human Influenza – 2015
http://ecdc.europa.eu/en/publications/_layouts/forms/Publication_DispForm.aspx?List=4f55ad51-4aed-4d32-b960-af70113dbb90&ID=1583

**Surveillance reports**
Date of publication: every Friday
http://ecdc.europa.eu/cdtr

**Scientific peer-reviewed publications**
Updated: September 2016
http://ecdc.europa.eu/en/publications/peer-reviewed/Pages/index.aspx

